# Supplementary material for: Comparative immunogenicity and reactogenicity of heterologous ChAdOx1-nCoV-19-priming and BNT162b2 or mRNA-1273-boosting with homologous COVID-19 vaccine regimens
Source: Nat Commun. 2022 Aug 11;13:4710. doi: 10.1038/s41467-022-32321-0 (PMC9366133; doi:10.1038/s41467-022-32321-0)
Supplement: Supplementary file 1 — Supplementary Information [file 41467_2022_32321_MOESM1_ESM.pdf]

Supplementary information

Comparative immunogenicity and reactogenicity of heterologous ChAdOx1 nCoV-19-priming and BNT162b2 or mRNA-1273-boosting with homologous COVID-19 vaccine regimens

Verena Klemis[1], Tina Schmidt[1], David Schub[1], Janine Mihm[2], Stefanie Marx[1], Amina Abu-Omar[1], Laura Ziegler[1], Franziska Hielscher[1], Candida Guckelmus[1], Rebecca Urschel[1], Stefan Wagenpfeil[3], Sophie Schneitler[4], Sören L. Becker[4], Barbara C. Gärtner[4], Urban Sester[2], and Martina Sester[1;*]

[1]Department of Transplant and Infection Immunology, [2]Department of Internal Medicine IV, [3]Institute for Medical Statistics, Epidemiology and Medical Informatics, Saarland University, Campus Homburg/Saar, [4]Institute of Medical Microbiology and Hygiene, Saarland University, 66421 Homburg, Germany.

*Correspondence: Martina Sester, PhD, Saarland University, Department of Transplant and Infection Immunology, Institutes of Infection Medicine, building 47, Kirrberger Straße, D-66421 Homburg, Germany; email: martina.sester@uks.eu

The supplement contains 6 Supplementary Tables and 3 Supplementary figures.

Supplementary Table 1: Correlations (coefficient, 95% CI and p-value) of immune parameters in individuals on the five regimens.

1° vaccine	ChAdOx		ChAdOx		ChAdOx		BNT		mRNA-1273	
2° vaccine	ChAdOx[1]		BNT[2]		mRNA-1273[3]		BNT		mRNA-1273	
	R (CI)*	P-value*	R (CI)*	P-value*	R (CI)*	P-value*	R (CI)*	P-value*	R (CI)*	P-value*
CD4 vs CD8	0.323 (0.072 – 0.536)	0.010	0.298 (0.053 – 0.509)	0.015	0.252 (0.053 – 0.431)	0.011	0.436 (0.147 – 0.656)	0.003	0.285 (0.021 – 0.512)	0.030
CD4 vs IgG	0.310 (0.058 – 0.525)	0.014	0.324 (0.082 – 0.530)	0.008	-0.110 (-0.304 – 0.093)	0.275	0.286 (-0.024 – 0.547)	0.063	0.207 (-0.062 – 0.448)	0.120
CD4 vs Neutralizing ab	0.325 (0.075 – 0.537)	<0.010	0.201 (-0.051 – 0.428)	0.106	0.072 (-0.131 – 0.269)	0.475	0.078 (-0.236 – 0.378)	0.619	0.201 (-0.068 – 0.443)	0.130
CD8 vs IgG	0.277 (0.022 – 0.498)	0.029	0.176 (-0.077 – 0.407)	0.158	-0.064 (-0.261 – 0.139)	0.527	0.438 (0.150 – 0.658)	0.003	0.313 (0.052 – 0.534)	0.017
CD8 vs Neutralizing ab	0.243 (-0.015 – 0.470)	0.057	0.006 (-0.243 – 0.255)	0.962	0.122 (-0.081 – 0.315)	0.225	0.360 (0.058 – 0.602)	0.018	0.264 (-0.001 – 0.495)	0.045
IgG vs Neutralizing ab	0.853 (0.763 – 0.910)	<0.0001	0.635 (0.459 – 0.763)	<0.0001	0.282 (0.086 – 0.457)	0.004	0.617 (0.381 – 0.778)	<0.0001	0.294 (0.031 – 0.519)	0.025

[1]Refers to ChAdOx1 nCoV-19 by AstraZeneca; [2]Refers to BNT162b2 by BioNTech/Pfizer, [3]Refers to mRNA-1273 by Moderna; *two-tailed Spearman correlation; source data are provided as a Source Data file; ab, antibody; R, correlation coefficient; CI, confidence interval.

Supplementary Table 2: Demographic and clinical characteristics of subgroups matched for age and gender.

1° vaccine	ChAdOx	ChAdOx	ChAdOx	BNT	mRNA-1273	
2° vaccine	ChAdOx[1]	BNT[2]	mRNA-1273[3]	BNT	mRNA-1273	
	n=40	n=40	n=40	n=40	n=40	p-value
**Years of age (mean±SD)**	50.0±11.8	49.2±9.4	49.8±10.7	51.0±17.9	48.6±13.1	0.942[§]
**Female gender, n (%)**	28 (70.0)	31 (77.5)	31 (77.5)	26 (65.0)	29 (72.5)	0.689[†]
**Weeks between 1° and 2° vaccination, (mean±SD)**	11.9±1.2	11.8±0.9	12.1±0.4	5.4±1.1	5.9±0.3	
**Analysis time [days after 2° vaccination], median (IQR)**	14 (2.75)	14 (1)	14 (1)	14 (2)	16 (2)	
**Differential blood cell counts**	n=40	n=39	n=40	n=38	n=39	
Leukocytes (cells/µl), median (IQR)	7000 (2500)	6400 (2000)	7000 (2750)	6000 (2750)	7700 (2900)	0.061[‡]
Granulocytes (cells/µl), median (IQR)	4064 (2070)	3819 (1279)	4083 (1736)	3415 (2229)	4617 (2379)	0.022[‡]
Monocytes (cells/µl), median (IQR)	556 (248)	578 (256)	578 (178)	479 (202)	539 (169)	0.118[‡]
Lymphocytes (cells/µl), median (IQR)	2100 (959)	2103 (828)	2243 (802)	2246 (803)	2150 (917)	0.544[‡]
CD3 T-cells (cells/µl), median (IQR)[#]	1526 (765)	1525 (655)	1489 (766)	1584 (666)	1636 (743)	0.920[‡]
CD4 T-cells (cells/µl), median (IQR)[#]	878 (600)	1024 (359)	996 (588)	1046 (522)	1144 (663)	0.904[‡]
CD8 T-cells (cells/µl), median (IQR)[#]	396 (219)	331 (235)	414 (304)	355 (252)	370 (155)	0.539[‡]
CD19 B cells (cells/µl), median (IQR)[#]	202 (132)	196 (141)	213 (164)	190 (168)	217 (155)	0.832[‡]
Plasmablasts (cells/µl), median (IQR)[#]	0.484 (0.611)	0.489 (0.835)	0.503 (0.592)	0.475 (0.468)	0.805 (0.801)	0.078[‡]

Demographic characteristics, vaccine-related data as well as information on differential blood counts and lymphocyte subpopulations are summarized for subgroups of the five different vaccination regimens. The 40 individuals per group have been chosen to be matched for age and gender to exclude age- and gender-related differences. [1]Refers to ChAdOx1 nCoV-19 by AstraZeneca; [2]Refers to BNT162b2 by BioNTech/Pfizer, [3]Refers to mRNA-1273 by Moderna; [#]B and T cell counts were calculated on 40 ChAdOx-ChAdOx, 39 ChAdOx-BNT, 40 ChAdOx-mRNA-1273, 36 BNT-BNT, and 39 mRNA-1273-mRNA-1273 vaccinated individuals, respectively. [§]Ordinary one-way ANOVA with Tukey´s multiple comparisons test. [†]X^2 test; [‡]two-sided Kruskal-Wallis test; source data are provided as a Source Data file.

Supplementary Table 3: Results of nonparametric regression analyses.

	**IgG**	**Neutralizing Ab**	**Spike CD4**	**Spike CD8**
**Confounders***	p-value	p-value	p-value	p-value
**Age**	0.739	0.439	0.258	0.779
**Gender**	0.218	1.000	0.469	0.714
**ChAdOx/BNT**	**<0.0001**	**<0.0001**	**<0.0001**	**<0.0001**
**ChAdOx/mRNA-1273**	**<0.0001**	**<0.0001**	**<0.0001**	**<0.0001**
**BNT/BNT**	**<0.0001**	**<0.0001**	**0.015**	0.890
**mRNA-1273/mRNA-1273**	**<0.0001**	**<0.0001**	**<0.0001**	0.782
**Interaction analyses#**				
**Age*ChAdOx/BNT**	0.221	0.947	0.140	0.060
**Age*ChAdOx/mRNA-1273**	0.589	0.281	0.095	0.203
**Age*BNT/BNT**	**0.003**	0.511	0.103	0.388
**Age*mRNA-1273/mRNA-1273**	**0.015**	0.268	0.185	0.499

The influence of age, gender, and the different vaccine regimens (using ChAdOx/ChAdOx as reference) on IgG levels, neutralizing antibody activity, and levels of spike-specific CD4 and CD8 T cells were analyzed using nonparametric regression. To determine whether age had an effect on immunological parameters within each vaccine group, interaction analyses were performed with the homologous ChAdOx vaccine group as a reference. Shown are the p-values determined by non-parametric regression analyses. *p-values refer to differences compared to ChAdOx/ChAdOx regimen; #confounding effects of age within each group was performed with ChAdOx/ChAdOx as a reference; source data are provided as a Source Data file.

Supplementary Table 4: Two-sided p-values from statistical comparisons of reactogenicity of the primary vaccines used for the first vaccination.

Vaccine regimens	**ChAdOx vs. BNT**	**ChAdOx vs. mRNA-1273**	**BNT vs. mRNA-1273**	**All groups**
Adverse events (general)[1]	**p<0.0001**	**p<0.0001**	p=0.064	**p<0.0001**
Pain[2]	p=0.485	p=0.109	p=0.076	**p<0.0001**
Swelling[2]	**p=0.0033**	p>0.999	**p=0.012**	**p<0.0001**
Antipyretic medication[3]	**p<0.0001**	**p<0.0001**	p>0.999	**p<0.0001**
Fever (<38.5°C)[3]	**p<0.0002**	**p<0.0001**	p>0.999	**p<0.0001**
Headache[3]	**p<0.0001**	**p<0.0001**	p=0.597	**p<0.0001**
Fatigue[3]	**p<0.0001**	**p=0.0004**	p=0.420	**p<0.0001**
Chills[3]	**p<0.0001**	**p<0.0001**	p=0.260	**p<0.0001**
GI effects[3]	**p=0.005**	**p=0.004**	p>0.999	**p=0.004**
Myalgia[3]	**p<0.0001**	**p<0.0001**	p=0.295	**p<0.0001**
Arthralgia[3]	**p<0.0001**	**p<0.0001**	p=0.506	**p<0.0001**

[1]Corresponding to differences in local, systemic or local and systemic adverse events as shown in figure 3a; [2]Corresponding to figure 3b; [3]Corresponding to figure 3c; ChaAOx refers to ChAdOx1 nCoV-19 by AstraZeneca; BNT refers to BNT162b2 by BioNTech/Pfizer, mRNA-1273 by Moderna; Fisher test has been used for comparison except for the comparison between all three primary vaccines where the X^2 test was used (last column). Two-sided tests were used. Statistically significant differences are indicated by bold type p-values; source data are provided as a Source Data file.

Supplementary Table 5: Two-sided p-values from statistical comparisons of reactogenicity of the regimens after the second vaccination.

Secondary vaccine regimen 1 *versus*	ChAdOx/ ChAdOx	ChAdOx/ ChAdOx	ChAdOx/ ChAdOx	ChAdOx/ ChAdOx	ChAdOx/ BNT	ChAdOx/ BNT	ChAdOx/ BNT	ChAdOx/ mRNA-1273	ChAdOx/ mRNA-1273	BNT/ BNT	All groups
Secondary vaccine regimen 2	ChAdOx/ BNT	ChAdOx/ mRNA-1273	BNT/ BNT	mRNA-1273/ mRNA-1273	ChAdOx/ mRNA-1273	BNT/ BNT	mRNA-1273/ mRNA-1273	BNT/ BNT	mRNA-1273/ mRNA-1273	mRNA-1273/ mRNA-1273	
Adverse events (general)[1]	**p<0.0001**	**p<0.0001**	p=0.134	**p<0.0001**	**p=0.001**	**p=0.002**	p=0.234	**p<0.0001**	p=0.213	**p=0.0008**	**p<0.0001**
Pain[2]	**p<0.0001**	**p<0.0001**	p=0.433	**p=0.0002**	p=0.380	**p=0.002**	p=0.821	**p<0.0001**	p=0.258	**p=0.009**	**p<0.0001**
Swelling[2]	p=0.802	**p=0.005**	p=0.193	**p=0.042**	**p=0.012**	p=0.120	p=0.080	**p=0.0002**	p=0.725	**p=0.002**	**p=0.0003**
Antipyretic medication[3]	**p=0.037**	**p<0.0001**	p=0.600	p=0.115	**p=0.024**	p=0.188	p=0.843	**p=0.0008**	**p=0.012**	p=0.350	**p<0.0001**
Fever (<38.5°C)[3]	p=0.058	**p=0.0003**	p=0.165	**p=0.011**	p=0.103	p=0.702	p=0.754	**p=0.060**	p=0.351	p=0.461	**p=0.005**
Headache[3]	**p=0.0008**	**p<0.0001**	p=0.207	**p=0.0003**	**p=0.004**	p=0.102	p=0.857	**p<0.0001**	**p=0.013**	p=0.061	**p<0.0001**
Fatigue[3]	**p=0.030**	**p<0.0001**	**p=0.043**	**p<0.0001**	**p=0.0002**	p>0.999	**p<0.0001**	**p=0.0015**	p=0.543	**p=0.0005**	**p<0.0001**
Chills[3]	**p=0.033**	**p<0.0001**	p=0.303	**p<0.0001**	p=0.072	p=0.522	**p=0.014**	**p=0.020**	p=0.353	**p=0.003**	**p<0.0001**
GI effects[3]	p=0.745	p=0.057	p=0.714	p=0.352	p=0.175	p>0.999	p=0.770	p=0.311	p=0.375	p=0.755	p=0.204
Myalgia[3]	**p=0.003**	**p<0.0001**	p=0.351	**p=0.0001**	**p=0.002**	p=0.102	p=0.340	**p<0.0001**	p=0.070	**p=0.013**	**p<0.0001**
Arthralgia[3]	p=0.139	**p=0.001**	p=0.734	p=0.125	p=0.109	p=0.096	p>0.999	**p=0.0012**	p=0.146	p=0.088	**p=0.001**

[1]Corresponding to differences in local, systemic or local and systemic adverse events as shown in figure 3a; [2]Corresponding to figure 3b; [3]Corresponding to figure 3c; ChAdOx refers to ChAdOx1 nCoV-19 by AstraZeneca; BNT refers to BNT162b2 by BioNTech/Pfizer, mRNA-1273 by Moderna; Fisher test has been used for comparison except for the comparison between all three primary vaccines where the X^2 test was used (last column). Two-sided tests were used. Statistically significant differences are indicated by bold type p-values; source data are provided as a Source Data file.

Supplementary Table 6: Antibodies for flow-cytometric analyses

Antigen	Conjugate	Clone	Isotype	Reactivity	Catalogue number
CD3	PerCP	SK7	IgG1 k	mouse anti-human	345766
CD4	APC-H7	SK3	IgG1 k	mouse anti-human	641398
CD8	V500	RPA-T8	IgG1 k	mouse anti-human	560774
CD8	PerCP	SK1	IgG1 k	mouse anti-human	345774
CD19	FITC	HIB19	IgG1 k	mouse anti-human	555412
CD27	APC	L128	IgG1 k	mouse anti-human	337169
CD38	PE	HB7	IgG1 k	mouse anti-human	345806
CD69	PE-Cy7	L78	IgG1 k	mouse anti-human	335792
CTLA-4	APC	BNI3	IgG2a k	mouse anti-human	555855
IFNγ	FITC	4S.B3	IgG1 k	mouse anti-human	554551
IgD	PE-Cy7	IA6-2	IgG2a k	mouse anti-human	561314
IL-2	PE	MQ1-17H12	IgG2a k	rat anti-human	559334
TNFα	V450	MAb11	IgG1 k	mouse anti-human	561311

All antibodies were purchased from BD.

Supplementary Figure 1

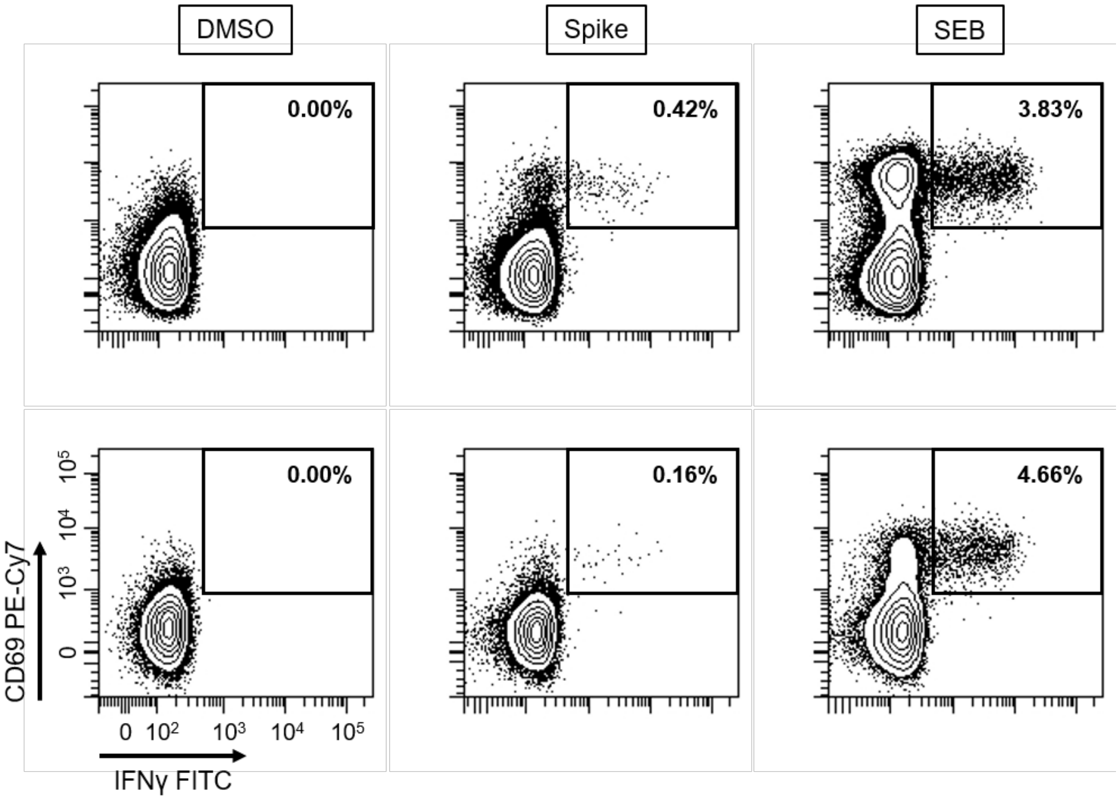

Supplementary Fig. 1: Representative flow-cytometric results of antigen-specific T cells after SARS-CoV-2 vaccination (mRNA-1273/mRNA-1273). Representative contour plots of CD4 and CD8 T cells after antigen-specific stimulation of a whole blood sample from a 49-years old individual 18 days after the second dose of the homologous mRNA-1273-vaccination regimen. Percentage of reactive (CD69+IFNγ+) cells among total CD4 (upper panel) or CD8 T cells (lower panel) were determined after stimulation with DMSO (negative control), overlapping peptides of SARS-CoV-2 spike protein or SEB (positive control). IFN, interferon; SEB, *Staphylococcus aureus* enterotoxin B.

Supplementary Figure 2

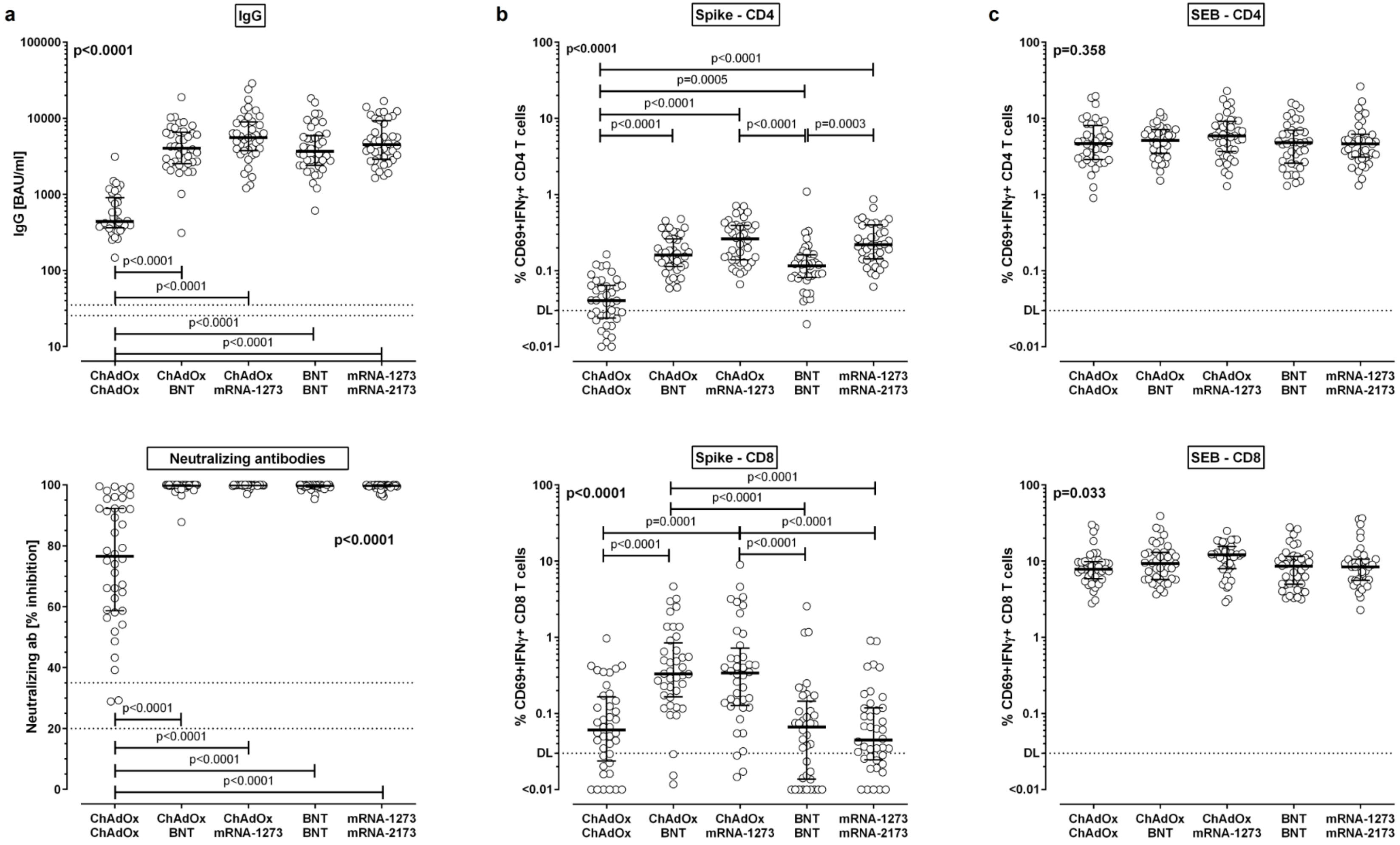

Supplementary Fig. 2: Immune responses against the SARS-CoV-2 spike protein after homologous COVID-19 vaccine regimens or heterologous ChAdOx-priming and BNT- or mRNA-1273-boosting in individuals matched for age and gender. Cellular and humoral immune parameters were analyzed two weeks post vaccination and compared between age- and gender-matched individuals (n=40 per vaccine group). **(a)** ELISA and surrogate neutralization assays were performed to quantify levels of spike-specific IgG and neutralizing antibodies. Intracellular cytokine staining after antigen-specific stimulation of whole blood samples allowed for flow-cytometrical determination of SARS-CoV-2 spike-specific **(b)** and SEB-reactive **(c)** CD4 and CD8 T-cell levels. Reactive cells were identified by co-expression of CD69 and IFNγ among CD4 or CD8 T cells and subtraction of background reactivity of respective negative controls. Bars represent medians with interquartile ranges. Differences between the groups were calculated using two-sided Kruskal-Wallis test with Dunn´s multiple comparisons post-test. Dotted lines indicate detection limits for antibodies in (a), indicating negative, intermediate and positive levels or levels of inhibition, respectively as per manufacturer's instructions, and detection limits for SARS-CoV-2-specific CD4 T cells in (b) and (c). Source data are provided as a Source Data file. IFN, Interferon; SEB, *Staphylococcus aureus* enterotoxin B.

Supplementary Figure 3

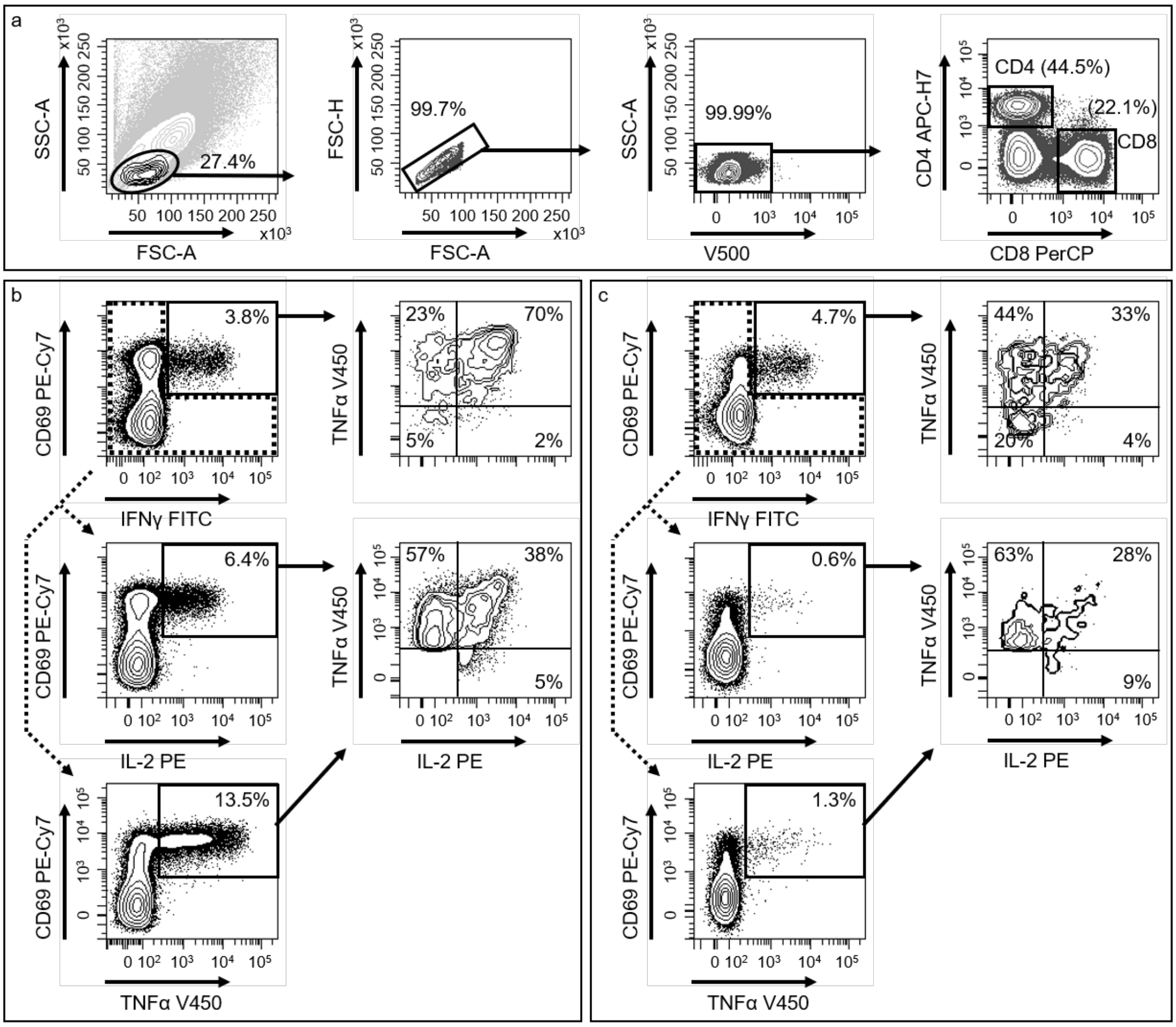

Supplementary Fig. 3: Gating strategy for analysis of antigen-specific T cells. (a) Lymphocytes were identified among total events by SSC-A and FSC-A signals and backgating of CD4 and/or CD8 positive cells. To improve the accuracy of the analysis, doublets were excluded by area and height signals of FSC and non-specific events were excluded using the V500 channel (not used for staining). Among single lymphocytes, T helper cells were determined as CD4-positive CD8-negative cells, whereas cytotoxic T cells were positive for CD8 and negative for CD4. Gating of cytokine-expressing antigen-specific T cells is shown in **(b)** for CD4 and **(c)** for CD8. CD69+IFNγ+ T cells were further subdivided into four subpopulations according to additional

expression of TNFα and IL-2. Using “NOT” Boolean Gating (dotted gates), T cells not being CD69+IFNγ+ were analyzed for CD69+IL-2+ and CD69+TNFα+. By using “OR” Boolean Gating, CD69+IL-2+ and CD69+TNFα+ were combined and divided into TNFα single, IL-2+TNFα+ or IL-2 single T cells. FSC, forward scatter; IFN, interferon; IL, interleukin; SSC, side scatter; TNF, tumor necrosis factor.
