# Supplementary material for: Comparative immunogenicity and reactogenicity of heterologous ChAdOx1-nCoV-19-priming and BNT162b2 or mRNA-1273-boosting with homologous COVID-19 vaccine regimens
Source: Nat Commun. 2022 Aug 11;13:4710. doi: 10.1038/s41467-022-32321-0 (PMC9366133; doi:10.1038/s41467-022-32321-0)
Supplement: Supplementary file 3 — Reporting Summary [file 41467_2022_32321_MOESM3_ESM.pdf]

nature portfolio

Corresponding author(s): Martina Sester

Last updated by author(s): 30.05.2022

Reporting Summary

Nature Portfolio wishes to improve the reproducibility of the work that we publish. This form provides structure for consistency and transparency in reporting. For further information on Nature Portfolio policies, see our Editorial Policies and the Editorial Policy Checklist.

Statistics

For all statistical analyses, confirm that the following items are present in the figure legend, table legend, main text, or Methods section.

n/a	Confirmed	
☐	☒	The exact sample size (*n*) for each experimental group/condition, given as a discrete number and unit of measurement
☐	☒	A statement on whether measurements were taken from distinct samples or whether the same sample was measured repeatedly
☐	☒	The statistical test(s) used AND whether they are one- or two-sided<br>*Only common tests should be described solely by name; describe more complex techniques in the Methods section.*
☐	☒	A description of all covariates tested
☒	☐	A description of any assumptions or corrections, such as tests of normality and adjustment for multiple comparisons
☐	☒	A full description of the statistical parameters including central tendency (e.g. means) or other basic estimates (e.g. regression coefficient) AND variation (e.g. standard deviation) or associated estimates of uncertainty (e.g. confidence intervals)
☐	☒	For null hypothesis testing, the test statistic (e.g. *F*, *t*, *r*) with confidence intervals, effect sizes, degrees of freedom and *P* value noted<br>*Give P values as exact values whenever suitable.*
☒	☐	For Bayesian analysis, information on the choice of priors and Markov chain Monte Carlo settings
☒	☐	For hierarchical and complex designs, identification of the appropriate level for tests and full reporting of outcomes
☐	☒	Estimates of effect sizes (e.g. Cohen's *d*, Pearson's *r*), indicating how they were calculated

Our web collection on statistics for biologists contains articles on many of the points above.

Software and code

Policy information about availability of computer code

Data collection	BD FACSdiva software 6.1.3<br>BD FACSuite software v1.4.0.7047
Data analysis	BD FACSdiva software 6.1.3<br>FlowJo software 10.6.2<br>GraphPad Prism software 9.0<br>SPSS V27 including an R 3.6 plug-in for non-parametric regression analysis

For manuscripts utilizing custom algorithms or software that are central to the research but not yet described in published literature, software must be made available to editors and reviewers. We strongly encourage code deposition in a community repository (e.g. GitHub). See the Nature Portfolio guidelines for submitting code & software for further information.

Data

Policy information about availability of data

All manuscripts must include a data availability statement. This statement should provide the following information, where applicable:

- Accession codes, unique identifiers, or web links for publicly available datasets
- A description of any restrictions on data availability
- For clinical datasets or third party data, please ensure that the statement adheres to our policy

Figures 2-5, Table 1, Supplementary Figure 2, and Supplementary Tables 1-5 have associated raw data. The data that support the findings of this study are available in a public repository.

Field-specific reporting

Please select the one below that is the best fit for your research. If you are not sure, read the appropriate sections before making your selection.

[x] Life sciences [ ] Behavioural & social sciences [ ] Ecological, evolutionary & environmental sciences

For a reference copy of the document with all sections, see nature.com/documents/nr-reporting-summary-flat.pdf

Life sciences study design

All studies must disclose on these points even when the disclosure is negative.

Sample size	observational study with sample size according to availability, real-world recruitment of vaccinated individual at Saarland University Medical campus (convenience sampling).
Data exclusions	Data from 1 individual with positive NCAP-IgG ratios were excluded from analyses based on a pre-established exclusion criterion. Exclusion of NCAP-IgG positive individuals was done as a history of infection is known to affect vaccine-induced immunity.
Replication	Experimental procedures were pre-established before. Sufficient cells and plasma volumes were available to be analysed on each sample. Within each sample, no replicates were performed due to limited sample material.
Randomization	not applicable; individuals were recruited in an observational study with convenience samples being collected.
Blinding	Individuals were recruited on the various regimens. During sample processing and analysis of primary data, the investigators were blinded to vaccine group allocation.

Reporting for specific materials, systems and methods

We require information from authors about some types of materials, experimental systems and methods used in many studies. Here, indicate whether each material, system or method listed is relevant to your study. If you are not sure if a list item applies to your research, read the appropriate section before selecting a response.

Materials & experimental systems

n/a	Involved in the study	
[ ]	[x]	Antibodies
[x]	[ ]	Eukaryotic cell lines
[x]	[ ]	Palaeontology and archaeology
[x]	[ ]	Animals and other organisms
[ ]	[x]	Human research participants
[x]	[ ]	Clinical data
[x]	[ ]	Dual use research of concern

Methods

n/a	Involved in the study	
[x]	[ ]	ChIP-seq
[ ]	[x]	Flow cytometry
[x]	[ ]	MRI-based neuroimaging

Antibodies

Antibodies used	anti-CD3 mouse anti-human (BD Biosciences, PerCP, clone SK7, cat. 345766, Dilution 1:25)<br>anti-CD4 mouse anti-human (BD Biosciences, APC-H7, clone SK3, cat. 641398, Dilution 1:100 for unstimulated samples and 1:33.3 for stimulated samples)<br>anti-CD8 mouse anti-human (BD Biosciences, V500, clone RPA-T8, cat. 560774, Dilution 1:100)<br>anti-CD8 mouse anti-human (BD Biosciences, PerCP, clone SK1, cat. 345774, Dilution 1:12.5)<br>anti-CD19 mouse anti-human (BD Biosciences, FITC, clone HIB19, cat. 555412, Dilution 1:40)<br>anti-CD27 mouse anti-human (BD Biosciences, APC, clone L128, cat. 337169, Dilution 1:200)<br>anti-CD28 mouse anti-human (BD Biosciences, purified, clone L293, cat. 348040, 1μg/ml)<br>anti-CD38 mouse anti-human (BD Biosciences, PE, clone HB7, cat. 345806, Dilution 1:20)<br>anti-CD49d mouse anti-human (BD Biosciences, purified, clone 9F10, cat. 555501, 1μg/ml)<br>anti-CD69 mouse anti-human (BD Biosciences, PE-Cy7, clone L78, cat. 335792, Dilution 1:33.3)<br>anti-IFNγ mouse anti-human (BD Biosciences, FITC, clone 4S.B3, cat. 554551, Dilution 1:100)<br>anti-IgD mouse anti-human (BD Biosciences, PE-Cy7, clone IA6-2, cat. 561314, Dilution 1:33.3)<br>anti-IL-2 rat anti-human (BD Biosciences, PE, clone MQ1-17H12, cat. 559334, Dilution 1:12.5)<br>anti-TNFα mouse anti-human (BD Biosciences, V450, clone Mab11, cat. 561311, Dilution 1:20)<br>anti-CTLA-4 mouse anti-human (BD Biosciences, APC, clone BNI3, cat. 555855, Dilution 1:50)
Validation	All antibodies were separately titrated in house to evaluate best concentrations for discrimination of negative and positive cells. Finally, panels including defined concentration of antibodies were tested together to confirm that identified antibody concentrations are also sufficient when compensations are required.

SK7: CD3 is intended for in vitro diagnostic use in the identification of cells expressing the CD3 antigen. Application: Flow cytometry; Source: https://www.bdbiosciences.com/en-de/products/reagents/flow-cytometry-reagents/clinical-diagnostics/single-color-antibodies-asr-ivd-ce-ivd/cd3-percp.345766

SK3: The CD4 antibody, clone SK3, is derived from the hybridization of mouse NS-1 myeloma cells with spleen cells from BALB/c mice immunized with human peripheral blood T lymphocytes. The CD4 antibody recognizes a 55-kilodalton (kDa) glycoprotein that is present on T-helper/inducer lymphocytes and monocytes. Application: Flow cytometry; Source: https://www.bdbiosciences.com/en-de/products/reagents/flow-cytometry-reagents/clinical-discovery-research/single-color-antibodies-ruo-gmp/apc-h7-mouse-anti-human-cd4.641398

RPA-T8: The RPA-T8 monoclonal antibody specifically binds to CD8 alpha (CD8α). CD8α is a type I transmembrane glycoprotein and a member of the immunoglobulin superfamily. CD8α is expressed by the majority of thymocytes, by subpopulations of αβ T cells and γδ T cells and by some NK cells. Cell surface CD8α is expressed either as a disulfide-linked homodimer (CD8αα) or as a heterodimer (CD8αβ) when disulfide-bonded to a CD8 beta chain (CD8β). CD8-positive αβ T cells coexpress both CD8αα homodimers and CD8αβ heterodimers whereas some γδ T cells and NK cells express CD8αα homodimers. CD8 plays important roles in T cell activation and selection. The extracellular IgSF domain of CD8α binds to a non-polymorphic determinant on HLA class I molecules (α3 domain) and enables CD8 to function as a co-receptor with MHC class I-restricted TCR during T cell recognition of antigen. The cytoplasmic domain of CD8α associates with Lck, a Src family protein tyrosine kinase that is involved in intracellular signaling. The RPA-T8 and HIT8a monoclonal antibodies are not cross-blocking. This clone has been reported to react with a subset of peripheral blood lymphocytes, but not monocytes nor granuloyctes, of baboon and both rhesus and cynomolgus macaque monkey. In general, a higher frequency of CD8+ and CD4+CD8+ lymphocytes are observed in non-human primates compared to normal human donors. Application: Flow cytometry (Routinely Tested); Source: https://www.bdbiosciences.com/en-de/products/reagents/flow-cytometry-reagents/research-reagents/single-color-antibodies-ruo/v500-mouse-anti-human-cd8.560774

SK1: CD8 is intended for in vitro diagnostic use in the identification of cells expressing CD8 antigen. Application: Flow cytometry; Source: https://www.bdbiosciences.com/en-de/products/reagents/flow-cytometry-reagents/clinical-diagnostics/single-color-antibodies-asr-ivd-ce-ivd/cd8-percp.345774

HIB19: The HIB19 monoclonal antibody specifically binds to the 95 kDa type I transmembrane CD19 glycoprotein. CD19 is expressed during all stages of B-cell maturation and differentiation, except on plasma cells. CD19 is also present on follicular dendritic cells. It is not found on T cells or on normal granulocytes. CD19 is a signal transduction molecule that regulates B cell development, activation, proliferation and differentiation. It associates with the complement receptor 2 (CD21), TAPA-1 (CD81), Leu 13, and/or MHC class II to form a signal transduction complex on the surface of B cells. Anti-CD19 clone HIB19 partially blocks the binding of clone B43, another CD19-specific monoclonal antibody. Application: Flow cytometry (Routinely Tested); Source: https://www.bdbiosciences.com/en-de/products/reagents/flow-cytometry-reagents/research-reagents/single-color-antibodies-ruo/fitc-mouse-anti-human-cd19.555412

L128: The CD27 antibody, clone L128, is derived from hybridization of mouse Sp2/0 cells with spleen cells from BALB/c mice immunized with activated peripheral blood lymphocytes. The CD27 antibody recognizes a 110–120-kilodalton (kDa) disulfide-linked homodimer comprised of two 55-kDa polypeptide chains. The CD27 antigen is a lymphocyte-specific member of the tumor necrosis factor receptor (TNFR) super family, which also includes nerve growth factor receptor, CD30, CD40, CD95 (Fas), CD120a, CD120b, CD134 (OX 40), and CD137. The CD27 antigen is also known as S152, T14, TNFRSF7, and Tp55. Application: Flow cytometry (RUO GMP); Source: https://www.bdbiosciences.com/en-de/products/reagents/flow-cytometry-reagents/clinical-discovery-research/single-color-antibodies-ruo-gmp/apc-mouse-anti-human-cd27.337169

L293: The CD28 antibody, clone L293, is derived from hybridization of Sp2/0-Ag14 mouse myeloma cells with spleen cells from BALB/c mice immunized with the HPB-ALL T-cell line. The CD28 antigen, a disulfide-linked homodimeric glycoprotein, Mr 44 kilodaltons (kd), is a cell-adhesion molecule (CAM) and functions as the ligand for CD80 (B7-1) and CD86 (B7-2) antigens, which are present on activated B lymphocytes, monocytes, and dendritic cells. Interaction of the CD28 antigen with CD80 or CD86 antigens, or both, co-stimulates CD2 and CD3 antigen/T-cell antigen receptor (TCR)–dependent T-cell–mediated proliferation and cytotoxicity. Application: Flow cytometry (RUO GMP); Source: https://www.bdbiosciences.com/en-de/products/reagents/flow-cytometry-reagents/clinical-discovery-research/single-color-antibodies-ruo-gmp/purified-mouse-anti-human-cd28.348040

HB7: CD38 is intended for in vitro diagnostic use in the identification of cells expressing CD38 antigen. Application: Flow cytometry; Source: https://www.bdbiosciences.com/en-de/products/reagents/flow-cytometry-reagents/clinical-diagnostics/single-color-antibodies-asr-ivd-ce-ivd/cd38-pe.345806

9F10: The 9F10 monoclonal antibody specifically reacts with the integrin α4 chain, that is expressed as a heterodimer with either of two β integrin subunits, β1 (CD29) or β7. The α4β1 integrin (VLA-4) is expressed on lymphocytes, monocytes, thymocytes, NK cells, and several B- and T-cell lines, and mediates binding to VCAM-1 (CD106) and the CS-1 region of fibronectin. The α4β7 integrin has a similar tissue distribution, except it is found on only a small subpopulation of thymocytes. Integrin α4β7 also binds fibronectin and VCAM-1, and has been shown in the mouse to preferentially bind the mucosal vascular addressin molecule, MAdCAM-1. This antibody is useful for studies of the expression by and function of cells that express α4 chain-containing integrins. This clone cross-reacts with a subset of peripheral blood lymphocytes, monocytes, and some granulocytes of baboon and both rhesus and cynomolgus macaque monkeys. The distribution on leukocytes is similar to that observed with human peripheral blood leukocytes. Application: Flow cytometry (Routinely Tested); Source: https://www.bdbiosciences.com/en-de/products/reagents/flow-cytometry-reagents/research-reagents/single-color-antibodies-ruo/purified-na-le-mouse-anti-human-cd49d.555501

L78: The CD69 antibody, clone L78, is derived from hybridization of mouse Sp2/0-Ag14 myeloma cells with lymph node cells from BALB/c mice immunized with a CD8+ alloantigen–directed cytotoxic T-lymphocyte (CTL) cell line. The CD69 antibody recognizes a very early human activation antigen that is a disulfide-bonded homodimer consisting of Mr 60-kilodalton (kDa) polypeptides with one or two N-linked oligosaccharides. Application: Flow cytometry (RUO GMP); Source: https://www.bdbiosciences.com/en-de/products/reagents/flow-cytometry-reagents/clinical-discovery-research/single-color-antibodies-ruo-gmp/pe-cy-7-mouse-anti-human-cd69.335792

4S.B3: The 4S.B3 monoclonal antibody specifically binds to interferon-γ (IFN-γ). The immunogen used to generate this hybridoma was partially purified human IFN-γ obtained from supernatants of human PBMC stimulated with Staphylococcus aureus. Interferon-γ (IFN-γ) is a potent multifunctional cytokine that is produced by several activated cell types including NK, NKT, CD4+TCRαβ+, CD8+TCRαβ+, and TCRγδ+ T cells. IFN-γ exerts its biological effects through specific binding to the high-affinity IFN-γ Receptor Complex comprised of IFN-γRα (CD119) and IFN-γRβ subunits. In addition to its antiviral effects, IFN-γ upregulates a number of lymphoid cell functions including the antimicrobial and antitumor responses of macrophages, NK cells, and neutrophils. In addition, IFN-γ can exert strong regulatory influences on the proliferation, differentiation, and effector responses of B cell and T cell subsets. These influences can involve IFN-γ's capacity to boost MHC class I and II expression by antigen-presenting cells as well as to direct effects on B cells and T cells themselves. Human IFN-γ is a 14-18 kDa glycoprotein containing 143 amino acid residues. Clone 4S.B3 also cross-reacts with a

cytoplasmic component of peripheral blood CD3+ lymphocytes of baboon, and both rhesus and cynomolgus macaque monkeys following five-hour treatment with phorbol myristic acetate (PMA) and Ca++ ionophore (A23187) in the presence of monensin. The staining pattern of 4S.B3 in CD3+ cells is similar to that observed with peripheral blood T lymphocytes from normal human donors. This reagent is useful for intracellular immunofluorescent staining for flow cytometric analysis to identify and enumerate IFN-γ + cells within a mixed cell population. Application: Intracellular staining (flow cytometry, Routinely Tested); Source: https://www.bdbiosciences.com/en-de/products/reagents/flow-cytometry-reagents/research-reagents/single-color-antibodies-ruo/fitc-mouse-anti-human-ifn.554551

IA6-2: The IA6-2 monoclonal antibody specifically binds to the heavy chain of human Immunoglobulin D (IgD). IgD is a member of the immunoglobulin superfamily that exists in type 1-membrane (mIgD) and soluble glycoprotein forms. mIgD is expressed on mature naïve B cells (along with membrane IgM) and serves as a B-cell receptor for antigen (BCR). In response to antigen binding, the mIgD BCR, in association with other signaling molecules including CD79a and CD79b, can transduce activating or tolerizing signals intracellularly into B lymphocytes. Application: Flow cytometry (Routinely Tested); Source: https://www.bdbiosciences.com/en-de/products/reagents/flow-cytometry-reagents/research-reagents/single-color-antibodies-ruo/pe-cy-7-mouse-anti-human-igd.561314

MQ1-17H12: The MQ1-17H12 monoclonal antibody specifically binds to the multifunctional cytokine, human Interleukin-2 (IL-2). IL-2 is produced by activated T cells and has multiple functions that can affect the growth, proliferation, differentiation and survival of many different target cell types including T cells, B cells, NK cells, monocytes and macrophages. The immunogen used to generate the MQ1-17H12 hybridoma was purified recombinant human IL-2 protein. The MQ1-17H12 antibody reportedly neutralizes the biological activity of human IL-2. Application: Intracellular staining (flow cytometry, Routinely Tested); Source: https://www.bdbiosciences.com/en-de/products/reagents/flow-cytometry-reagents/research-reagents/single-color-antibodies-ruo/pe-rat-anti-human-il-2.559334

MAb11: The MAb11 monoclonal antibody specifically binds to human tumor necrosis factor (TNF, also known as TNF-α) protein. TNF is an efficient juxtacrine, paracrine and endocrine mediator of inflammatory and immune functions. It regulates the growth and differentiation of a variety of cell types. TNF is cytotoxic for transformed cells when in conjunction with IFN-γ. It is secreted by activated monocytes/macrophages and other cells such as B cells, T cells and fibroblasts. The immunogen used to generate the MAb11 hybridoma was recombinant human TNF. The MAb11 antibody has been reported to crossreact with Rhesus Macaque TNF. Application: Intracellular staining (flow cytometry, Routinely Tested); Source: https://www.bdbiosciences.com/en-de/products/reagents/flow-cytometry-reagents/research-reagents/single-color-antibodies-ruo/v450-mouse-anti-human-tnf.561311

BNI3: The BNI3 monoclonal antibody specifically binds to the human cytolytic T lymphocyte-associated antigen (CTLA-4), also known as CD152. CTLA-4 is transiently expressed on activated CD28+ T cells and binds to CD80 and CD86 present on antigen presenting cells (APC) with high avidity. This interaction appears to deliver a negative regulatory signal to the T cell. Recent reports indicate that CTLA-4 is also expressed on B cells when cultured with activated T cells, suggesting a role for CTLA-4 in the regulation of B-cell response. Immobilized BNI3 antibody enhances T-cell proliferation induced by antibody-mediated crosslinking of CD3 and CD28. Recent studies have shown that CD152 can be expressed by regulatory T (Treg) cells. After cellular fixation and permeabilization, the BNI3 antibody can stain intracellular CD152 expressed in T cells including Treg cells. Clone BNI3 was studied in the VI Leukocyte Typing Workshop. Source: APC Mouse Anti-Human CD152 (bdbiosciences.com). Clone BNI3 also cross-reacts with a subset of peripheral blood lymphocytes of baboon, and both rhesus and cynomolgus macaque monkeys, following Concanavalin A (Con A) treatment. The distribution of BNI3+ cells following activation is similar to that observed with peripheral blood lymphocytes from normal human donors

Human research participants

Policy information about studies involving human research participants

Population characteristics	Immunocompetent individuals with no known history of SARS-CoV-2 infection were included in the study. Individuals were enrolled after having received a primary vaccination with the ChAdOx1 nCoV-19 or one of the mRNA-vaccines (BNT162b2 or mRNA-1273) and before receiving the secondary vaccine. Five groups were included comprising homologous regimens comprising either ChAdOx1 nCoV-19 (n=62, 52.5±10.9 years of age, 41 females, 21 males), BNT162b2 (n=43, 52.9±18.7 years of age, 26 females, 17 males) or mRNA-1273 (n=59, 41.9±15.2 years of age, 39 females, 20 males), or a heterologous vaccine regimen comprising a ChAdOx1 nCoV-19 priming dose followed by secondary vaccination with BNT162b2 (n=66, 45.0±10.9 years of age, 53 females, 13 males) or with mRNA-1273 vaccine (n=101, 38.2±13.7 years of age, 82 females, 19 males). One female on an mRNA-1273/mRNA-1273 regimen was excluded from final analysis due to positive IgG towards NCAP.
Recruitment	Prospective recruitment; healthy immunocompetent individuals were invited to participate in the study (most were health care workers at Saarland University Medical Center); all participants gave written informed consent; matching for time (14 days) after second vaccine dose with an interval of 13-18 days tolerated. All individuals were enrolled prior to the second vaccine as convenience sampling, and assignment to the vaccine groups were not randomized but determined by current guidelines. This may cause a bias in age, which was further addressed by subgroup analyses and non-parametric regression analyses.
Ethics oversight	Ethikkommission der Ärztekammer des Saarlandes

Note that full information on the approval of the study protocol must also be provided in the manuscript.

Flow Cytometry

Plots

Confirm that:

- [x] The axis labels state the marker and fluorochrome used (e.g. CD4-FITC).
- [x] The axis scales are clearly visible. Include numbers along axes only for bottom left plot of group (a 'group' is an analysis of identical markers).
- [x] All plots are contour plots with outliers or pseudocolor plots.
- [x] A numerical value for number of cells or percentage (with statistics) is provided.

Methodology

Sample preparation	Whole blood analysis
Instrument	BD FACS Canto II, BD FACSLyric
Software	BD FACSdiva software 6.1.3<br>BD FACSuite software v1.4.0.7047<br>FlowJo software 10.6.2<br>GraphPad Prism software 9.0<br>SPSS V27 including an R 3.6 plug-in for non-parametric regression analysis
Cell population abundance	All blood samples were tested without further processing (i.e. no purification or cell sorting)
Gating strategy	Gating strategies are outlined in dedicated figure.<br><br>Gating of antigen-reactive T cells:<br>Lymphocytes were identified among total events by backgating of CD4 and/or CD8 positive cells combined with signals for size (FSC) and granularity (SSC). Hight and area signals of FSC were used to exclude doublets. CD4 T-cells were identified among single cells by CD4 positive and CD8 negative signals. Likewise, CD8 T-cells were defined as T-cells being CD8 positive and CD4 negative. Antigen-reactive cells were identified as CD4 or CD8 T-cells co-expressing the activation marker CD69 and the cytokines IFNγ, IL-2 and/or TNFα. Boundaries between "cytokine-negative" and "cytokine-positive" was defined using negative control stimulations.<br>For additional analysis of cytokine expression profiles, NOT Boolean Gating was used to identify all CD4 T-cells that were not CD69+IFNγ+. Among these cells CD69+IL-2+ and CD69+TNFα+ CD4 T-cells were gated and combined using OR Boolean Gating. CD69+IL-2+ and/or CD69+TNFα+ CD4 T-cells were divided into IL-2 single, TNFα single or IL-2+TNFα+ cells.<br>Gating of T cells and plasmablasts:<br>Lymphocytes were identified among total events by signals for size (FSC) and granularity (SSC). Hight and area signals of FSC were used to exclude doublets. CD3 positive T-cells were identified among single lymphocytes and further subclassified in CD4 and CD8 T-cells. Among single lymphocytes, B cells were identified by CD19 expression and subclassified in naïve, non-switched memory and switched memory B cells according to expression of IgD and CD27. Plasmablasts were identified by high expression of CD38 among switched memory B cells.

- [x] Tick this box to confirm that a figure exemplifying the gating strategy is provided in the Supplementary Information.
